# Micro-Photographs in Histology

**Published:** 1877-02

**Authors:** 


*Micro-Photographs in Histology.*—Monthly numbers of this work, containing Normal and Pathological Photographs, by Carl Seiler, M. D., in connection with J. Gibbons Hunt, M. D., and J. G. Richardson, M. D., are now being issued by J. H. Coates & Co., Publishers, Philadelphia.

These Photographs are obtained directly from the microscopic objects by the aid of photography, and printed from the negative by a certain and reliable process, so that the pictures are faithfully copied, and give exact impressions of the specimens as they really are. These Micro-Photographs are of the greatest service to those whose time or opportunity will not permit them to use the microscope for themselves.

Each number contains four plates, two normal and two pathological, with descriptive text. Subscription price six dollars per annum.

